# Diversity and Inclusivity in Rheumatology Publications

**DOI:** 10.1002/acr2.11721

**Published:** 2024-08-01

**Authors:** Amr H. Sawalha, Kelli D. Allen, Candace H. Feldman, S. Sam Lim, Andras Perl, Daniel H. Solomon, Edith M. Williams

**Affiliations:** ^1^ University of Pittsburgh School of Medicine Pittsburgh Pennsylvania; ^2^ University of North Carolina at Chapel Hill, and Durham VA Health Care System Durham North Carolina; ^3^ Brigham and Women's Hospital and Harvard Medical School Boston Massachusetts; ^4^ Emory University School of Medicine Atlanta Georgia; ^5^ State University of New York Upstate Medical University, Norton College of Medicine Syracuse New York; ^6^ University of Rochester School of Medicine and Dentistry Rochester New York

With a steadfast advancement in scientific methods, technologies, and bioinformatics tools, an exciting era of exponential growth in medical knowledge is upon us. In rheumatology, we are enthusiastic about the potential benefits that scientific developments can bring to our patients. We predict significant improvements in quality of life, better diagnostics and therapeutics, and potentially the eradication of some chronic autoimmune diseases in the near future.

As we progress with our scientific and clinical investigations, our responsibility to advance medical knowledge must be interconnected with a strong commitment to diversity, equity, and inclusivity. It is far too common that research in medicine, including in rheumatology, lacks focus on underrepresented racial and ethnic groups disproportionately impacted by the disease conditions investigated. The injustice of excluding certain groups from the opportunity to participate in research significantly limits the applicability of the new knowledge gained to guide evidence‐based treatment decisions and advocate for equitable distribution of resources for our patients. Embracing diversity, equity, and inclusion is paramount and should be applied early during study design, throughout the research process, and in publication.

At the journals of the American College of Rheumatology, our duty is to ensure that every individual is treated with utmost respect, regardless of ethnicity, race, geographic origin, sex, age, gender identity, or any other factor. We call upon our authors, reviewers, and editors to champion diversity, equity, and inclusion in every aspect of study design and analyses, reporting outcomes, and dissemination of medical knowledge.

In this editorial, we present overarching guidelines to ensure that our journals adhere to our commitment to accurate reporting, diversity, and inclusivity. We urge authors, reviewers, and editors of our journals to carefully scrutinize textual and graphical elements in submitted manuscripts to ensure these guidelines are honored. The overall goal is to improve the accuracy and inclusivity of reported research findings and avoid erroneous attribution of findings, for example, of inequities by race or ethnicity, to biological and genetic variables.^1^, ^2^

It is crucial to emphasize that “race” and “ethnicity” are terms that should not be used to reflect biological variability and are, therefore, not interchangeable with “ancestry” or “genetic background.” Indeed, the National Academies of Sciences, Engineering, and Medicine recommends that race should not be used as proxy for genetic ancestry or genetic variability.^3^ This view is also shared by the American Medical Association and is reflected by the guidelines of the *Journal of the American Medical Association*.^1^ Race and ethnicity are often surrogates for nongenetic factors, such as environmental exposures and social determinants of health.^2^

Research studies involving human patients should clearly state the methodology used to ascertain race and ethnicity, including whether self‐reporting was used and if an open question or a form with a choice from a set category was used. If the latter, then the choices included on the forms should be listed in the Methods or in supplementary material. To standardize terminology, we favor using the National Institutes of Health guidelines for race and ethnicity reporting (NOT‐OD‐15‐089),^4^ although we realize that these guidelines might continue to change. Where pertinent, authors are encouraged to include self‐identified countries of origin.

The terms “people of color” or “minorities” are discouraged.^1^ Similarly, terms that collectively refer to racial or ethnic groups, such as “non‐White” and “mixed race,” or certain terms related to color, such as “brown” and “yellow,” should not be used. The use of the term “Caucasian” should not indicate “White” or individuals of European descent. Terms used to refer to race and ethnicity should not be used in noun form (eg, Asians, Blacks, Hispanics, or Whites); instead, they should be used as adjectives such as Asian Americans, Black patients, Hispanic individuals, or White children. Moreover, terms referring to race, ethnicity, or tribe should be capitalized, underscoring respect for racial, ethnic, and cultural identity. When citing previous research that uses discouraged terms, it is essential to provide the rationale for retaining these terms in the manuscript if it is necessary to ensure accuracy. We encourage active engagement with relevant populations before and during the course of the study to foster inclusivity, enhance diversity in study recruitment, and ensure accurate and sensitive reporting.

Appropriate terms should be used when referring to individuals with disabilities. We encourage adhering to the Disability Inclusive Language Guidelines set forth by the United Nations Office at Geneva.^5^ In general terms, reference to disability should only be made when it is relevant to the research question or scientific findings in the manuscript. Terms such as “handicap,” “retarded,” “crippled,” or “disabled” should be avoided. Instead, “a person with disability” should be used, and the type of disability can be specified as needed (eg, “a person with a physical or an intellectual disability”). Similarly, terms such as “wheelchair‐bound” or “confined to a wheelchair” should be replaced by terms such as “using a wheelchair.” Furthermore, terms such as “suffer from,” “afflicted by,” “stricken by,” or “troubled with” are discouraged and should be replaced by simply saying “having” a specific condition.

The distinction between reporting sex and gender should be clear.^6^ The term sex should be used when referring to biological factors, and gender should be used when referring to gender identity. Further, sex or gender designation should be used as adjectives rather than nouns. For example, “female patients” rather than “females.” Information about gender identity and sexual orientation should be reported when relevant to the study question and the findings being described.^6^ We encourage using terms suggested by the Centers for Disease Control and Prevention to describe gender identity and sexual orientation.^7^ We also encourage using precise terms when describing sex and gender, and avoiding terms such as “the opposite sex” or “the opposite gender.” When possible, using gender‐neutral terms are preferred (eg, “Humankind” instead of “Mankind”).

Finally, authors, reviewers, and editors should ensure that the limitations of research findings from a narrow representation of racial, ethnic, and ancestral groups, or from studies lacking gender diversity, are transparently acknowledged. The lack of generalizability of such findings should be considered for discussion.

In conclusion, the American College of Rheumatology is committed to upholding the highest standards of diversity, inclusivity, and science in our publications. Embracing these standards and guiding principles will ensure accurate scientific reporting and maintain respect and dignity of all study participants. The editorial teams of our journals will continue to ensure that this guidance is followed.

## AUTHOR CONTRIBUTIONS

All authors contributed to at least one of the following manuscript preparation roles: conceptualization AND/OR methodology, software, investigation, formal analysis, data curation, visualization, and validation AND drafting or reviewing/editing the final draft. As corresponding author, Dr Sawalha confirms that all authors have provided the final approval of the version to be published, and takes responsibility for the affirmations regarding article submission (eg, not under consideration by another journal), the integrity of the data presented, and the statements regarding compliance with institutional review board/Helsinki Declaration requirements.

## Supporting information


Disclosure form
